# Special Issue: A Tale of Genes and Genomes

**DOI:** 10.3390/genes12050774

**Published:** 2021-05-19

**Authors:** Mario Ventura, Francesca Antonacci

**Affiliations:** Department of Biology, University of Bari ‘Aldo Moro’, 70125 Bari, Italy

Variability is the source on which selective pressure acts, allowing genome evolution and adaptation. Large-scale comparative sequencing promises to reconstruct the evolutionary history of the human genome and to highlight the functional genetic differences between human and other species. Thanks to the recent advances in genome sequencing, assembling and studying complex regions of the genome enriched in segmental duplications and repetitive elements is not a limited process anymore. This is extremely important, on the one hand, in measuring the contribution of the emergence of novel genes in species diversification and, on the other hand, in evaluating the effect of evolutionary genomic rearrangements in human disease recurrence.

This Special Issue consists of nine original research articles and two reviews covering various aspects of genome organization and gene family evolution. Two original research publications are focused on abiotic stress-related genes such as *BoPUB* and *NHX* genes and their expression and adaptation in plants. In particular, Hu and colleagues performed genomewide identification and evolutionary analysis of the U-box protein family of *Brassica oleracea* (*BoPUB* genes) [1]. The *BoPUB* genes underwent a family expansion from the *Arabidopsis thaliana* to the *B. oleracea* genomes. RNA-seq transcriptome data of different pollination times revealed spatiotemporal expression specificity of the *BoPUB* genes. The study provides valuable information indicating that the *BoPUB* genes not only participate in the abiotic stress response but also are involved in pollination. Akram et al. instead performed a genomewide analysis of *NHX* genes in *Gossypium barbadense* in comparison with *Gossypium hirsutum* [2]. The authors investigated phylogenetic relationships, transcript expression under salt stress in different tissues, chromosomal location, and gene structures. The authors showed that through a systematic analysis of all the members of the *NHX* gene family, they can compare the gene regulation, expression pattern, and eventually their biological functions in cotton, providing valuable information explaining the molecular mechanism of Na+ transport for further functional studies of Gossypium *NHX* genes.

Kim and colleagues’ study focused on the rapid evolutionary radiation of the pufferfish (Takifugu species) in marine ecosystems [3]. The genus Takifugu has undergone explosive radiation relatively recently and contains a subset of closely related species with a scale-loss phenotype. The authors showed that the scale-loss phenotype of two Takifugu species, *T. pardalis* Temminck & Schlegel and *T. snyderi* Abe, is largely controlled by an overlapping genomic segment (QTL). The QTL region contains no known genes responsible for the evolution of the scale-loss phenotype in other fishes. The genes used for the scale-loss phenotypes in the two Takifugu species are likely the same, but they are different from genes with the same function in distantly related fishes. The authors identified in the QTL region *Fgfrl1* a gene predicted to function in a pathway known to regulate bone/scale development. Since *Fgfr1a1*, another member of the Fgf signaling pathway, has been implicated in the scale loss/scale shape in fish distantly related to Takifugu, the authors suggested that the convergence of the scale-loss phenotype may be constrained by signaling modules with conserved roles in scale development.

In recent years, technical advances in genome sequencing have greatly enhanced our ability to investigate the genetic differences within and between human populations and other species. Shen and colleagues developed QuicK-mer2, a method for studying copy number variation that enables the rapid construction of paralog-specific copy number maps from short-read sequence data [4]. The authors applied the approach to newly released sequence data from the 1000 Genomes Project, constructed paralog-specific copy number maps from 2457 unrelated individuals, and uncovered copy number variation of paralogous genes. They identified nine genes where none of the analyzed samples have a copy number of two and 92 genes where the majority of the samples have a copy number higher than two, and described rare copy number variation affecting multiple genes at the *APOBEC3* locus.

In their review, Lallemand and colleagues described the evolutionary processes allowing the formation of duplicated genes and the various bioinformatic approaches that can be used to identify them in genome sequences [5]. Indeed, these approaches differ according to the underlying duplication mechanism. Hence, understanding the specificity of the duplicated genes of interest is a great asset for tool selection and should be taken into account when exploring a biological question.

Using single-molecule, real-time sequencing, in silico analyses, and molecular cytogenetics, Maggiolini and colleagues characterized the structure, copy number, and chromosomal distribution of the *POTE* genes, a primate-specific gene family expressed in the prostate, ovary, and testis, as well as in several cancers, including breast, prostate, and lung cancers [6]. This gene family maps within human and primate segmental duplications with a copy number ranging from 2 to 14 in different species. The authors de novo sequenced and assembled a *POTE* tandem duplication in marmoset that is misassembled and collapsed in the reference genome, thus revealing the presence of a second *POTE* copy. The study provides comprehensive insights into the evolutionary dynamics of the primate-specific *POTE* gene family, involving gene duplications, deletions, and long interspersed nuclear element (LINE) transpositions to explain the actual repertoire of these genes in human and primate genomes.

Structural variants (SVs), including deletions and inversions, comprise a larger proportion of genetic differences between and within species, making them an important yet understudied source of trait divergence. Recent comparisons of short- and long-read sequencing technologies using benchmark human genomic datasets revealed that multiple genomes and combinatorial platforms are required for comprehensive SV discovery. Soto and colleagues performed long-range optical mapping and Oxford Nanopore Technologies long-read sequencing to characterize the structural variant landscape of two Pan troglodytes versus individuals [7]. These new datasets have allowed for a more comprehensive assessment of deletions and inversions in the chimpanzee genome. When compared with published whole-genome screens using orthogonal approaches, this approach validated existing variants and discovered many new variants. Knowing that SVs often alter gene functions and regulation, the authors characterized the association of the discovered SVs on differences in gene regulation and chromatin organization between human and chimpanzee, identifying a number of events that likely contribute to chimpanzee-specific differences.

Batcher and colleagues successfully used whole-genome sequence data to identify novel polymorphic retrocopies of the *FGF4* gene in canids [8]. Gene retrocopies, often referred to as processed pseudogenes, often go unidentified or misidentified by common variant calling methods. The authors suggested that their approach could be applied to all genes to identify other polymorphic gene retrocopies that may play an important role in both breed health and phenotypic variation across dogs.

Scardino and colleagues investigated the history of human chromosomes using comparative cytogenetic approaches [9]. Human chromosome 13 has previously been shown to be conserved as a single syntenic element in the ancestral primate karyotype. The authors used fluorescence in situ hybridization (FISH) to characterize the high level of rearrangements in *Saguinus oedipus*, *Callithrix argentata*, and *Alouatta belzebul*, providing insight into the evolution of human chromosome 13. Furthermore, a review of previous cytogenomic literature data on chromosome 13 evolution in eutherian mammals showed a complex origin of the eutherian mammal ancestral karyotype, which has still not been completely clarified.

Insights into the evolution of the modern eukaryotic cells come from the study of Diroma and colleagues [10]. By applying an ad hoc computational pipeline based on the MToolBox software, the authors reconstructed mtDNA genomes in single cells using whole-genome and exome sequencing data obtained by different amplification methodologies as well as data from scATAC-seq in which mtDNA sequences are expected as a byproduct of the technology. The authors showed that assembled mtDNAs, with the exception of those reconstructed by MALBAC and DOP-PCR methods, are quite uniform and suitable for genomic investigations, enabling the study of various biological processes related to cellular heterogeneity, such as tumor evolution, neural somatic mosaicism, and embryonic development.

Finally, Antonacci and colleagues told us the tale of the T cell receptor (TR) loci in the adaptive immune response [11]. T lymphocytes are the principal actors of vertebrates’ cell-mediated immunity. They can recognize an unlimited number of foreign molecules through their antigen-specific heterodimer receptors (TRs). A great plasticity of the gene organization within the TR loci exists among species, and this is mainly due to gene duplication and somatic rearrangement during T cell differentiation. Antonacci and colleagues reported the most recent findings on the genomic organization of TRG loci in mammalian species in order to show differences and similarities. The comparison revealed remarkable diversification of both the genomic organization and gene repertoire across species, but also unexpected evolutionary conservation, which highlights the important role of T cells in immune response.

Overall, the papers in this Special Issue cover various aspects of genome organization and gene family evolution and show the importance of combining the use of different methodological approaches to provide an overview of the dynamic and plasticity of genome architecture in both the animal and plant kingdoms.
